# Intramedullary Fixation With a Short Nail in a Young Patient Presenting With a Pathological Proximal Femur Fracture

**DOI:** 10.5435/JAAOSGlobal-D-21-00055

**Published:** 2021-06-02

**Authors:** Marcos Roberto González, Mayte Bryce-Alberti, Ty Subhawong, Muhammad Hakim, Andrew Rosenberg, Juan Pretell-Mazzini

**Affiliations:** From the Facultad de Medicina Universidad Peruana Cayetano Heredia, Lima, Peru (Dr. González, Dr. Bryce-Alberti); the Department of Radiology, Division of Musculoskeletal Radiology, University of Miami Miller School of Medicine (Dr. Subhawong); the Department of Bone and Soft Tissue Pathology, Miami Miller School of Medicine (Dr. Hakim, Dr. Rosenberg); and the Department of Orthopaedics, Division of Musculoskeletal Oncology, University of Miami Miller School of Medicine, Miami, FL (Dr. Pretell-Mazzini).

## Abstract

An 18-year-old man presented with a pathological fracture of the right proximal femur. Desmoplastic fibroma was diagnosed through histological studies. Surgical management involved extended intralesional curettage and fracture stabilization by open reduction with intramedullary nailing, using a short Gamma nail. At 42-month follow-up, the patient presented no limitations or recurrence. Internal fixation after prior intralesional curettage is a valid treatment strategy for pathological fractures in young patients. A short nail was chosen to prevent direct tumor cell seeding throughout the femur and future recurrence. Fracture consolidation was achieved because of the healing potential of a young patient.

Desmoplastic fibroma (DF) is a rare primary intraosseous tumor that represents 0.3% of benign bone tumors and 0.06% of all bone neoplasms. Most affected patients are young, and the tumor is most commonly located in the jaw and the metaphysis of long bones.^1,2^ Although histologically benign, this tumor is locally aggressive, recurs often, and rarely undergoes malignant transformation.^3-5^

When located in the femur, an infrequent location, most patients manifest slowly progressive pain, and only a small minority present with a pathological fracture.^6^ Treatment strategies for this patient cohort are variable, and the optimal approach is still unclear. Physicians must choose an intervention that balances the oncological and functional outcomes,^7^ considering the possibility of recurrence and a need for subsequent surgeries.

Our study presents the case of an 18-year-old otherwise healthy man who consulted to our musculoskeletal oncology service after being diagnosed with a pathological fracture of the right proximal femur undergoing a functional and oncological treatment approach.

The patient was informed that data concerning this case would be submitted for publication and he provided consent.

## Case Report

A previously healthy 18-year-old man presented to our emergency department with right hip pain and was unable to ambulate following a low-energy impact while playing basketball. He reported feeling a sudden, inaudible “pop” immediately after the hit. On admission, the patient complained of excruciating right hip pain followed by occasional numbness and tingling in the right lower extremity.

Physical examination showed tenderness to palpation of the right proximal thigh area. The right lower extremity had intact skin, and no distal neurovascular compromise was detected. The rest of the examination was noncontributory. Medical history was normal, except for the presence of right groin pain approximately 4 to 6 months ago. At the time, an ultrasonography failed to identify any pathology.

**Figure 1:**
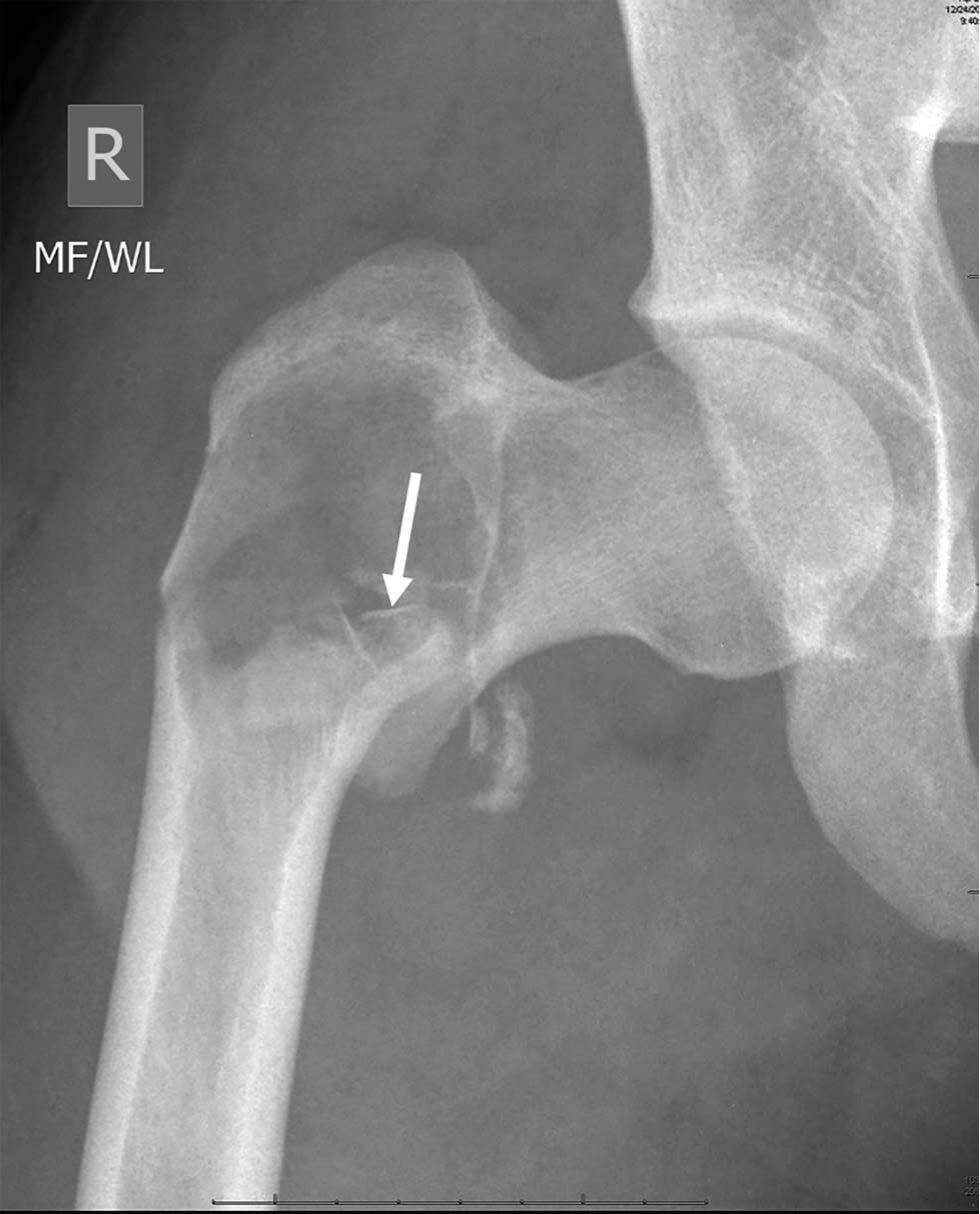
AP radiograph of the right hip at presentation demonstrates a pathological fracture through a sharply marginated lytic lesion in the intertrochanteric region of the right femur. Note small bony fracture fragments (arrow) in the dependent portion of the lytic lesion, simulating the “fallen fragment” sign seen in unicameral bone cysts.

**Figure 2:**
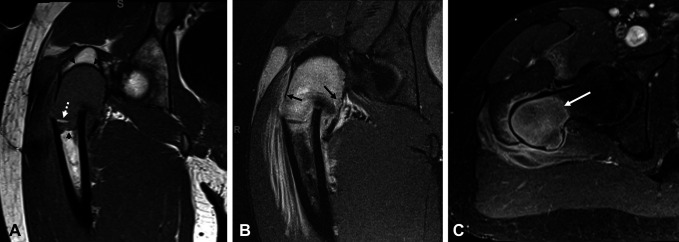
MRI of the right hip with and without contrast: **A**, Coronal T1-weighted MRI demonstrates the intertrochanteric pathological fracture with varus angulation. The underlying tumor is well-defined by a hypointense rim at both its superior and inferior margins (arrowheads). Intratumoral bone fragments can also be appreciated (dashed arrow). **B**, Coronal fat-suppressed T2-weighted image demonstrates the T2 hyperintense tumor in the proximal femur, with extensive surrounding bone marrow edema and hemorrhage caused by the pathological fracture. Note the marked thinning and endosteal scalloping along both the medial and lateral cortices (arrows). **C**, Axial contrast-enhanced fat-suppressed T1-weighted MRI shows somewhat heterogeneous enhancement in the intraosseous tumor, above the level of the fracture. Note the well-defined boundary with normal marrow in the mid-cervical region (arrow).

**Figure 3:**
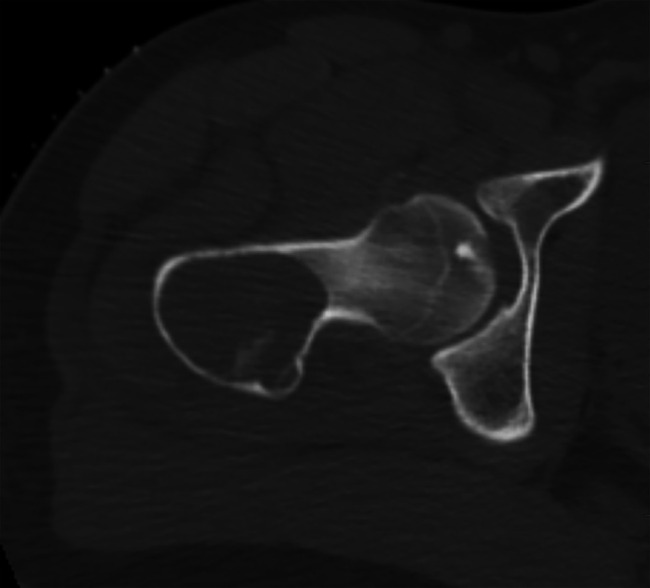
Axial CT above the pathological fracture demonstrates the lytic lesion with well-defined margins, high-grade endosteal scalloping, and posterior cortical buckling.

On admission, AP and lateral right hip radiographs demonstrated (Figure 1) a pathological intertrochanteric femur fracture associated with a radiolucent lesion measuring 5.4 × 5.6 cm and varus angulation. The margin has a narrow zone of transition and medially and, to a lesser extent, superiorly, has a fine sclerotic rim. There appears to be a groundglass matrix so that a benign fibro-osseous tumor is a reasonable thought. Magnetic resonance imaging of the right hip (Figure 2, A–C) demonstrated a hypointense T1-weighted, hyperintense T2-weighted lesion in the proximal femur with postcontrast heterogeneous enhancement. CT (Figure 3) showed a lytic lesion with well-defined margins, high-grade endosteal scalloping, and posterior cortical buckling.

**Figure 4:**
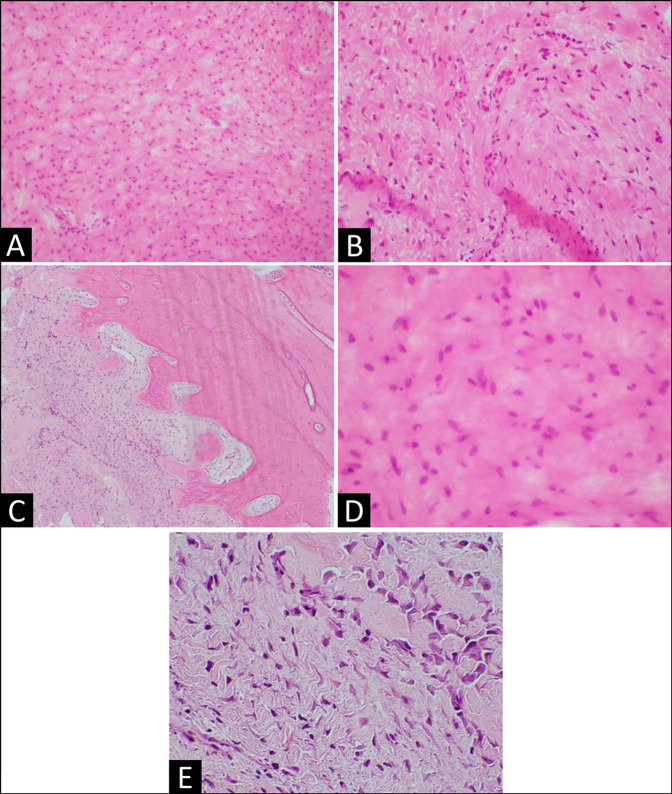
Histopathological features of desmoplastic fibroma: **A**, Desmoplastic fibroma–spindled cells with bland small nuclei, evenly dispersed in the collagenous stroma (low power). **B**, Slender spindled cells set within abundant eosinophilic collagen matrix (low power). **C**, Spindled cell proliferation accompanied by collagenous stroma-desmoplastic fibroma infiltrating bone (low power). **D**, Spindled cells with indistinct cytoplasmic borders and bland ovoid nuclei with smooth contours show finely dispersed chromatin. Cells appear to merge with the intercellular collagenous matrix. No mitoses present (high power). **E**, Interface of desmoplastic fibroma and bone-spindled cells with elongated nuclei (lower left) enmeshed between wavy collagen fibers abut large polyhedral osteoblasts (upper right) within and surrounding the nascent osteoid (high power).

The patient was transferred to our musculoskeletal oncology service for definitive management. Radiological differential diagnoses included fibrous cortical defect, fibrous dysplasia, bone cyst, giant cell tumor of bone, chondroblastoma (greater trochanter apophysis), and metastasis less likely. A CT-guided bone biopsy was conducted, and histological features confirmed the diagnosis of DF (Figure 4). CT images of the chest, abdomen, and pelvis showed no evidence of primary tumor.

**Figure 5:**
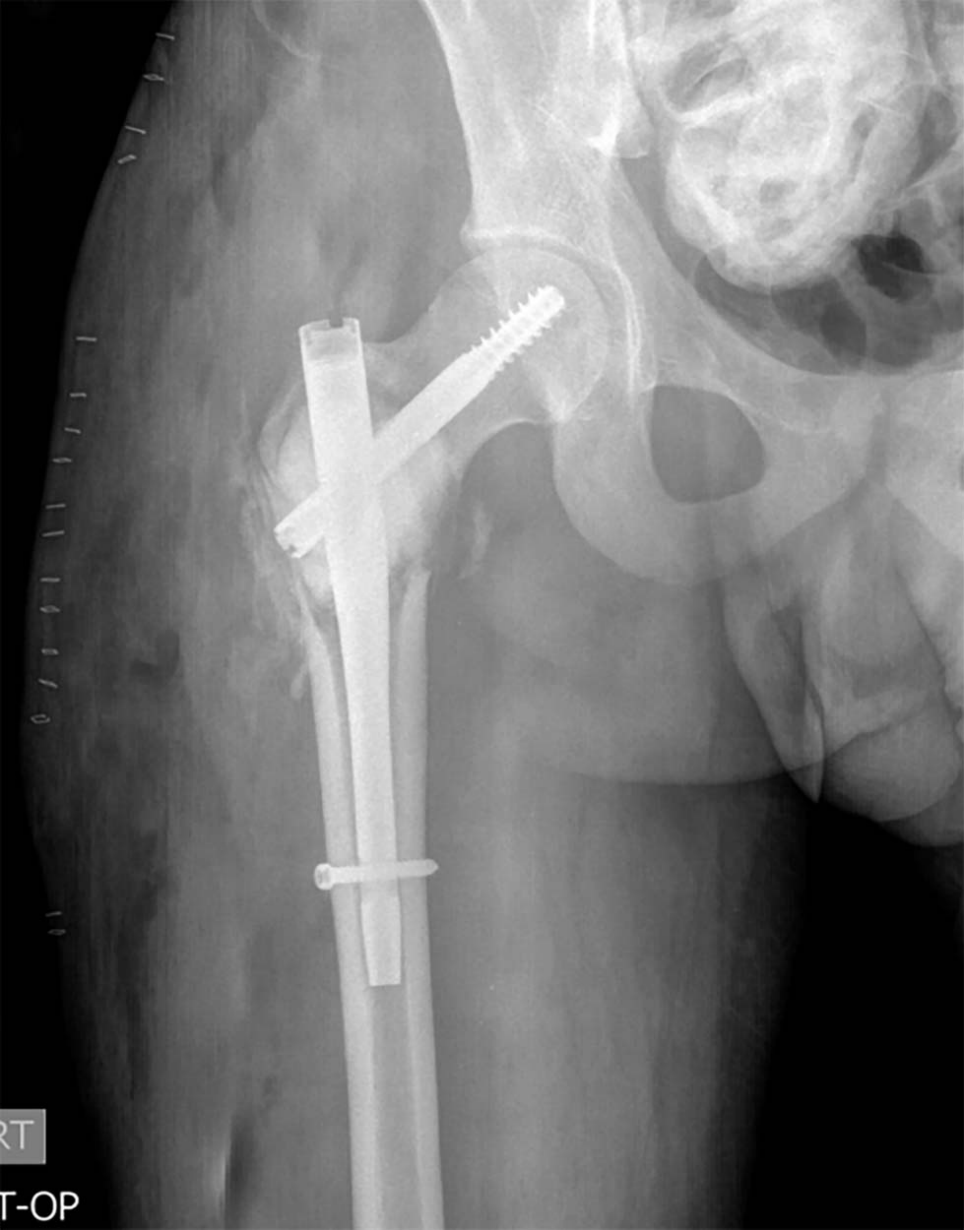
Postoperative AP radiograph of the right hip after intralesional treatment with curetting and bone grafting, fracture reduction, and internal fixation with a short intramedullary nail.

Considering this tumor's benign nature and the patient's young age, we chose to surgically manage the case through extended intralesional curettage. Proximal and distal fracture sites were curetted, and afterward, phenol 50% and 95% alcohol were applied to the area of the tumor. The fracture was stabilized by open reduction with intramedullary (IM) nailing, using a short Gamma nail (Stryker). Afterward, Wright Medical calcium sulfate paste was applied, and a cortical powder allograft bone graft was introduced through the fracture line (Figure 5).

**Figure 6:**
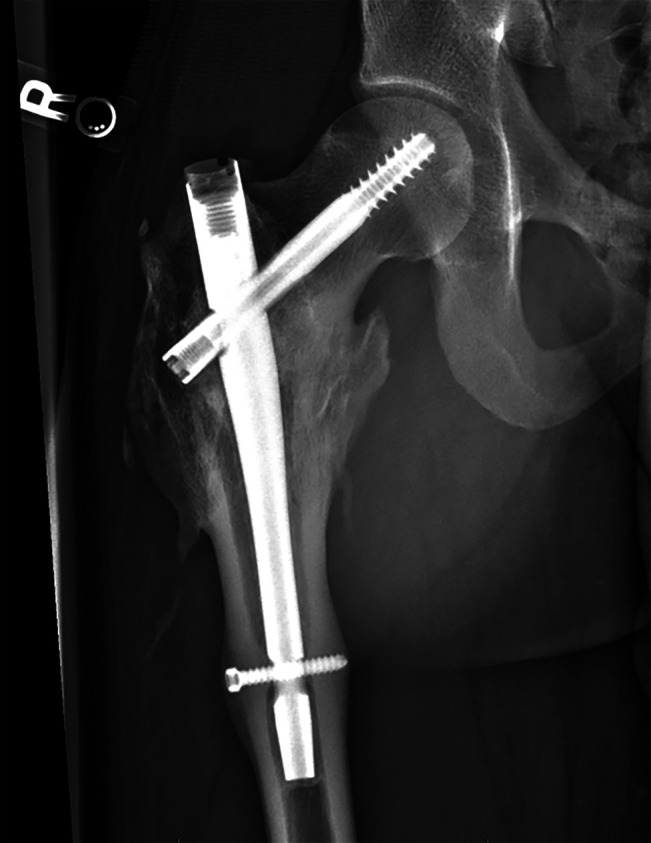
Three-year follow-up AP radiograph demonstrates complete incorporation of the graft and healing of the fracture, with no evidence of recurrent disease or implant loosening.

The postoperative plan involved a progressive weight-bearing protocol over the right lower extremity with full weight bearing at 3 months, anticoagulation with enoxaparin for 4 weeks, and initiation of physical therapy within the subsequent couple of days. At 42-month follow-up, the patient is able to walk and play basketball again without limitations. On physical examination, the right hip has full range of motion in comparison with the contralateral side, and plain radiographs showed evidence of consolidation without tumor recurrence (Figure 6).

## Discussion

DF is a rare bone tumor that manifests as a slowly growing tumor with lytic appearance. Despite being histologically benign, it may demonstrate local aggression and can infiltrate adjacent soft tissues. The lesion arises in the medullary canal and may progress with or without cortical destruction. Periosteal reaction and mineralization is uncommon. In addition, there are high tumor recurrence rates, and in rare cases, malignant transformation after surgical treatment is of concern.^8^

DF bears radiological resemblance with various benign and malignant lesions. CT may detect matrix formation and cortical involvement, and magnetic resonance imaging can aid in further assessment of intraosseous extension and soft-tissue component.^3^ Ultimately, diagnosis is dependent on histological characteristics. Histology of DF is similar to that of a desmoid tumor, its soft-tissue counterpart. The lesion is composed of spindle-shaped cells surrounded by an abundant collagenous background. Nuclear atypia and mitotic activity is limited. Although immunohistochemistry lacks specificity, the tumor may express vimentin and smooth muscle actin.^1,2^

Literature concerning surgical management of femoral DF is scarce. We conducted a review of all published case reports and found that to this date, only 15 additional cases have been published in the English literature (Table 1). In addition, only one of these patients reported a pathological fracture associated with DF. The most common location for the tumor within the femur was the distal region (10 patients); four patients had a lesion located in the proximal femur and one in the midshaft region.

**Table 1:** Literature Review of Published Surgical Management of Desmoplastic Fibroma in the Femur

Reference	Journal	Year of Publication	Years of Age at Diagnosis	Lesion Location in the Femur	Fracture	Intervention	Outcome	Follow-up Period
Stevens et al^6^	*The Journal of Bone and Joint Surgery*	2019	24	Mid-shaft	Yes, mid-shaft fracture	En bloc excision, intramedullary nail, and exchange nail	No recurrence	7 yr
Xu et al^13^	*Medicine (Baltimore)*	2018	25	Distal	No	Wide surgical resection and allogeneic graft	No recurrence	1 yr
Tanwar et al^14^	*Indian Journal of Surgical Oncology*	2018	65	Proximal	No	Excision, extended curettage, and fibular grafts	No recurrence	4 yr
			31	Distal	No	Excision, extended curettage, and cementing		10 wk
Gong et al^15^	*Chinese Medical Journal*	2018	46	Proximal	No	Curettage	No recurrence	1 yr
Ishizaka et al^1^	*Journal of Orthopaedic Science*	2018	32	Proximal	No	Wide resection, reconstruction with recycled bone, and fibula graft	No recurrence	8 mo
Gong et al^3^	*Oncology Letters*	2015	21	Proximal	No	Curettage, bone grafting, and cementation	No recurrence, pathological fracture 4 mo after surgery	28 yr
Yokouchi et al^16^	*Oncology Letters*	2014	26	Distal	No	Extended curettage, heat ablation, and artificial bone grafting	No recurrence	12 yr
Gao et al^17^	*Oncology Letters*	2013	66	Distal	No	Radical resection and internal fixation	No recurrence	5 yr
Min et al^18^	*Annals of Diagnostic Pathology*	2010	41	Distal	No	Curettage, mass resection, and allograft reconstruction	Malignant transformation (unspecified)	Unspecified
Rastogi et al^19^	*Joint Bone Spine*	2008	24	Distal	No	Extended curettage, autologous cancellous bone graft, and fibular bone grafting	No recurrence	6 yr
Takazawa et al^5^	*Journal of Orthopaedic Science*	2003	37	Distal	No	Curettage and bone grafting	Malignant transformation (osteosarcoma)	16 yr
Böhm et al^9^	*Cancer*	1996	43	Distal	No	Excision and arthrodesis	No recurrence	3 yr
Clayer et al^20^	*Clinical Orthopaedics and Related Research*	1994	17	Distal	No	Distal intralesional curettage, proximal en bloc resection, and allograft replacement	No recurrence	3 yr
Bertoni et al^21^	*The Journal of Bone and Joint Surgery*	1984	24	Distal	No	Wide excision and endoprosthesis	No recurrence, complicated with infection that lead to amputation	35 yr

Proposed surgical approaches for DF include extended intralesional curettage or wide resection followed by reconstruction. Böhm et al^9^ reported a recurrence rate of 55% for simple curettage, and 25% of recurrences in the extremities were ultimately treated with amputation. Nishida et al^10^ reported 5 cases of patients with DF treated with aggressive curettage and found no recurrence; however, this could be attributed to the absence of extraosseous tumor extension in all patients. Wide local excision is indicated in some patients, but associated morbidity and relatively short life span of endoprosthesis relatively to the life expectancy of young patients with benign entities pose a hurdle for its use in young patients such as ours.

Specific recommendations for managing pathological fractures associated with femoral DF are unavailable. Nevertheless, internal fixation with IM nails or plates after prior intralesional curettage to minimize tumor cell spreading is usually the treatment choice for pathological fractures in young patients without extensive bone damage.^7^ In addition, because young patients are expected to live for many years after the procedure, failure of proximal femoral internal fixation can be further treated with revision internal fixation with or without bone grafting or hip arthroplasty.^11^

In older patients with no good bone stock, arthroplasty in terms of proximal and total femoral arthroplasties is a more feasible option than in young patients. In this patient cohort, IM nailing is reserved for diaphyseal pathological fractures of the femur, while those of the femoral head and neck are treated with hemiarthroplasty.^11^

When subtrochanteric pathological fractures present with severe bone loss, they are usually treated with resection followed by endoprosthetic reconstruction.^7^ Although our patient presented with poor bone stock, we did not pursue this strategy. Instead, after an extended intralesional curettage, we stabilized the fracture by open reduction and IM nailing using a short Gamma nail (Stryker) and bone grafting with allograft and calcium sulfate substitute. We thought that bone stock regeneration was possible because of our patient's young age and health. Also, using an allograft potentially avoids donor-site morbidity (autograft), and the use of calcium sulfate substitutes has been associated with rapid biological integration and an early return to activities of daily living, with no composite-related complications.^12^ A short nail was chosen over a long nail to prevent direct tumor cell seeding throughout the femur with potential future recurrence in areas that would require resection and reconstruction with a total femoral arthroplasty. Biomechanically, a long nail in this fracture pattern provides greater stability that in turn allows fracture consolidation. In our case, fracture healing occurred with a short nail because it provided enough stability to result in fracture healing and consolidation of the defect associated with stress on the distal portion of the nail, as it can be seen in plain radiographs (Figure 6). These demonstrate thickening of the shaft at that level, which is explained by the Wolff's law premise that mechanotransduction leading to remodeling can overtime strengthen bone and allow it to resist increasing loads.^13^ The nail did not fail also because of the healing potential of a young patient as in our case.

Finally, the recurrence rate for DF has historically been deemed high. Reported values show it to be as high as 48% related mainly to cases in which curettage is performed.^9^ Conversely, our literature review (Table 1) evidenced that not a single patient experienced recurrence. The average follow-up time was 12 years and 2 months, and among the 14 patients included (one case report did not specify the follow-up time), two presented with malignant transformation to sarcoma. This case report had a follow-up period of 42 months. In our case, the patient regained and maintained complete functionality with fracture consolidation and no evidence of radiological recurrence.
